# Platelet-Rich Plasma as an Autologous and Proangiogenic Cell Delivery System

**DOI:** 10.1155/2017/1075975

**Published:** 2017-08-06

**Authors:** Jessica Zahn, Markus Loibl, Christoph Sprecher, Michael Nerlich, Mauro Alini, Sophie Verrier, Marietta Herrmann

**Affiliations:** ^1^AO Research Institute Davos, Clavadelerstrasse 8, 7270 Davos Platz, Switzerland; ^2^Department of Trauma Surgery, University Medical Center Regensburg, Regensburg, Germany

## Abstract

Angiogenesis is a key factor in early stages of wound healing and is crucial for the repair of vascularized tissues such as the bone. However, supporting timely revascularization of the defect site still presents a clinical challenge. Tissue engineering approaches delivering endothelial cells or prevascularized constructs may overcome this problem. In the current study, we investigated platelet-rich plasma (PRP) gels as autologous, injectable cell delivery systems for prevascularized constructs. PRP was produced from human thrombocyte concentrates. GFP-expressing human umbilical vein endothelial cells (HUVECs) and human bone marrow-derived mesenchymal stem cells (MSCs) were encapsulated in PRP gels in different proportions. The formation of cellular networks was assessed over 14 days by time-lapse microscopy, gene expression analysis, and immunohistology. PRP gels presented a favorable environment for the formation of a three-dimensional (3D) cellular network. The formation of these networks was apparent as early as 3 days after seeding. Networks increased in complexity and branching over time but were only stable in HUVEC-MSC cocultures. The high cell viability together with the 3D capillary-like networks observed at early time points suggests that PRP can be used as an autologous and proangiogenic cell delivery system for the repair of vascularized tissues such as the bone.

## 1. Introduction

Angiogenesis is crucial for healing and regeneration of vascularized tissues such as the bone [1]. In this process, new capillaries sprout from preexisting vessels to support expanding vascular networks. In the case of large musculoskeletal defects, the surrounding tissue is usually damaged, which may pose a problem since an adequate supply of oxygen and nutrients at the injury site is essential for proper healing [2]. To date, this constitutes still a clinical challenge, because on the one hand, the distance which can be reached by angiogenic sprouting is limited, and on the other hand, the ingrowth of new vessels is a slow process with 5 *μ*m/h [3].

Replacement of the damaged tissue might be a suitable option but is often constricted by the availability of transplantable graft material, for example, bone grafts. Cell-based tissue engineering strategies may overcome these problems. However, it has been shown that the ingrowth of vessels into grafts, as well as the repopulation with endothelial cells into grafts, is not rapid enough to ensure a sufficient blood supply of the grafted cells/tissue [4–6]. In contrast, preformed networks could be linked to existing vessels within 2–4 days [6, 7]. These findings strongly suggest the use of prevascularized constructs [8].

Cell-based prevascularization of tissue-engineered constructs, using endothelial cells [9–12], endothelial progenitor cells (EPCs) [13–15], or microvascular fragments [16], showed promising results, supporting a fast anastomosis to the host vascular system. Interestingly, it has variously been shown that mural cells, such as mesenchymal stem cells (MSCs), or fibroblasts further promote the vascularization process [9, 10, 13, 15].

The culture and expansion of EPCs and endothelial cells, for example, HUVECs, require proangiogenic growth factors. Such recombinant growth factors are expensive and discussed controversially [17]. The supplementation of growth medium with autologous growth factors would be desirable for clinical application. Platelets are part of the blood and contain more than 5000 proteins. About 300 of the contained proteins, especially growth factors and cytokines, are released upon activation [18–20]. The release of cytokines, chemokines, and growth factors induces proliferation and activation of the cells that are involved in wound healing such as fibroblasts, neutrophils, monocytes, smooth muscle cells, and MSC [21]. In addition, our previous work has demonstrated that platelet-derived growth factors can be efficiently used as culture supplement for EPCs [15, 22, 23].

In the last two decades, the treatment with platelet-related plasma (PRP), defined as suspended plasma which contains at least 200,000–1,000,000 platelets/*μ*l suspended plasma [24], is upcoming. PRP can be produced by commercial centrifuges and is used for various clinical applications in the field of orthopedics, for example, cartilage repair [25, 26], ligament healing [27–29], and tendon healing [30–32]. Although PRP provides a spectrum of growth factors released by platelets, there is no common definition of PRP preparations and often PRP products have not been systematically tested in clinical trials [33–35], both hindering comparisons between different studies.

Thrombin and/or calcium chloride are commonly used to activate platelets in PRP [36], which provokes the release of biomolecules and cleavage of fibrinogen resulting in the formation of a fibrin gel. Therefore, activated PRP can be used as an autologous hydrogel [37–39]. Taking advantage of an autologous system, PRP has been applied for various *in vitro* and *in vivo* studies. Due to its high content of growth factors, such as platelet-derived growth factor (PDGF), transforming growth factor *β* (TGF-*β*), and insulin-like growth factor (IGF) [40], PRP has been used for various applications of musculoskeletal regeneration, orthopedics, and the treatment of ischemic diseases [13, 41–44]. We have recently demonstrated that PRP gels can be used as a cell delivery system for MSCs [33]. In this study, we showed that PRP sustained viability and promoted proliferation of encapsulated MSCs in a platelet concentration-dependent manner.

Based on promising preliminary data [45], the aim of the present study was to investigate the angiogenic properties of PRP gels and to evaluate whether PRP can be used as an autologous delivery system for prevascularized structures supporting early neovascularization.

## 2. Methods

### 2.1. Bone Marrow- (BM-) Derived Mononuclear Cell (MNC) Isolation and Cell Culture

BM aspirates were obtained from vertebra of patients undergoing orthopedic surgery after informed consent and approval by the local ethics committee (EK Regensburg 12-101-0127). Mononucleated cells (MNCs) were isolated from BM aspirates (*n* = 4) by density centrifugation with Ficoll (Histopaque®-1077, Sigma). MNCs were seeded in tissue culture flasks at a density of 5 × 10^4^ cells/ cm^2^ in *α*MEM (Gibco) containing 10% FBS (Seraplus, Pan) and 5 ng/ml bFGF (R&D Systems). After 4 days in culture, nonadherent hematopoietic cells were removed. Mesenchymal stem cells (MSCs) selected by adherence to cell culture plastic were further expanded with a change of medium every 3 days. Cells were passaged when 80% confluency was reached, detached using 1% trypsin-EDTA and reseeded at a density of 3 × 10^3^ cells/cm^2^. All MSCs were used from passages 2 to 4.

GFP-expressing HUVEC cells (HUVEC-GFP, Angio-Proteomie) were cultured on coated plates (Speed Coating Solution, PELObiotech) in complete EGM-2 growth medium (Lonza) at a seeding density of 5 × 10^3^ cells/cm^2^ and subcultured upon 80% confluency. Cells were used in passages 7–9.

### 2.2. Cell Labeling of MSCs

For live cell imaging, MSCs were labeled with PKH26® Red Fluorescent Cell Linker Kit for General Cell Membrane Labeling (Sigma Aldrich) prior encapsulation in PRP gels. After detaching and counting of cells, the desired amount of cells were pipetted in a new falcon tube, washed with serum-free *α*MEM and centrifuged at 400*g* for 5 min. Cells were resuspended in Diluent C (Sigma) and filtered using a 40 *μ*m cell strainer, and dye working solution was added (2 *μ*l PKH26 Red/1 × 10^6^ MSCs) and incubated for 5 min at room temperature. The labeling reaction was stopped by addition of FBS (SeraPlus), and cells were washed three times with medium and the cell count was determined.

### 2.3. Preparation of PRP

PRP was produced from human leukocyte-depleted thrombocyte concentrates (blood bank, Kantonsspital Graubünden, Chur, Switzerland) obtained by apheresis containing 1000 × 10^3^ platelets/*μ*l and less than 2 leukocytes/*μ*l. In order to reach an approximately 10-fold increased platelet concentration compared to physiological blood concentration (150–300 × 10^3^ platelets/*μ*l), the platelet concentrates were centrifuged at 2000*g* for 20 min. The resulting pellet was resuspended in half of the original volume of platelet-depleted plasma, resulting in PRP with 2000 × 10^3^ platelets/*μ*l. PRP was homogenized by sonication for 15 min and stored at −20°C until use. PRP samples (*n* = 3) were pooled and randomly matched to normalize for any donor-specific influences.

### 2.4. Encapsulation of Cells in PRP Gels

For the incorporation of cells into PRP gels, PRP aliquots (pool of 3 donors) were thawed. Cells were seeded in PRP gels at a density of 2.5 × 10^3^ cells per *μ*l gel. For time-lapse microscopy, a gel volume of 20 *μ*l was seeded in *μ*-slides (*μ*-Slide Angiogenesis, Ibidi); for histology and gene expression analysis, 120 *μ*l gels were prepared in 96-well plates. Monocultures of each cell type and cocultures were performed as follows: 100% MSCs (100 M), 100% HUVECs (100 H), 75% HUVECs–25% MSCs (75 HM), and 50% HUVECs–50% MSCs (50 HM). Cells were resuspended in PRP and gelation induced by addition of human thrombin (Tisseel, Baxter, final concentration 5 U/ml) and incubation at 37°C for 15–30 min. All gels were cultured under static conditions at 37°C and 5% CO_2_ in EGM-2 growth medium (Lonza) with 5 *μ*M *ε*-aminocaproic acid (Sigma).

### 2.5. Microscopy and Image Analysis

The *μ*-slides with PRP gels were placed in an onstage incubator linked to an EVOS™ FL Auto Cell Imaging System (ThermoFisher Scientific). The cellular network formation capacity of HUVECs and MSCs in different proportions was analyzed using time-lapse microscopy. Regions of interest were defined before starting of the time lapse to allow tracking of individual cells. Pictures were taken every three hours for 14 days and medium changed every two days. At the end of the experiment, gels were imaged using a LSM 510 confocal imaging system, equipped with an argon and a HeNe1 laser, and mounted on an Axiovert200m microscope with ZEN black software (all Zeiss). Gels were imaged using the 10x and 20x objective at 1272 × 1272 and 2028 × 2028 pixel, respectively.

**Figure 1:**
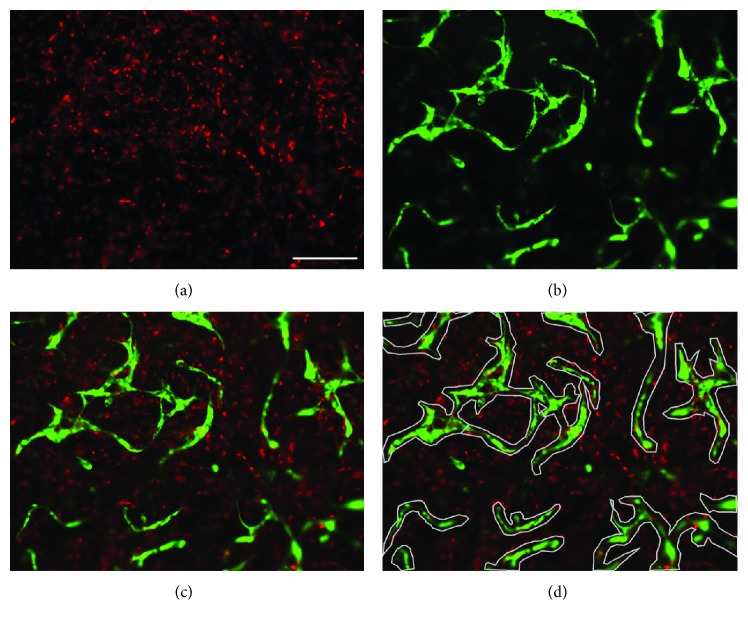
Network analysis. Image analysis method of PRP gels (seeding density: 2.5 × 10^3^ cells/*μ*l gel) with encapsulated PKH26 Red-labeled MSCs and GFP-HUVECs (green). Shown are representative pictures of a 50% HUVEC–50% MSC coculture after 14 days of culture. (a) Red-labeled MSCs, (b) GFP-labeled HUVECs, and (c) combined image. (d) Tubular structures are marked with the polygons (grey). Scale bar = 200 *μ*m.

Image analysis of cellular networks was performed as described before [46]. In brief, the PKH26 Red-labeled MSCs (Figure 1(a)) were imaged with the RFP filter and GFP-HUVECs (Figure 1(b)) with the GFP filter (both ThermoFisher Scientific). The combined images of both fluorescences (Figure 1(c)) generated with the EVOS FL Auto Cell Imaging System (ThermoFisher Scientific) were imported in Axiovision Software (version 4.9.1, Zeiss). For quantification of cellular networks, any tubular structures were marked with a polygon area (Figure 1(d)) and the area measured. The image analysis was performed with the KS400 software (Zeiss) and a custom-made macro. An individual threshold was set for the images of the red-labeled MSCs and GFP-HUVECs, and the area of the respective fluorescent dye within the region of interest was calculated.

### 2.6. Gene Analysis

Two sample gels were taken and pooled at different time points for gene expression analysis: day 0 (d0), day 7 (d7), and day 14 (d14). For RNA extraction, pooled gels were lysed in 1 ml TriReagent (Sigma) and supplemented with 5 *μ*l Polyacryl Carrier (Molecular Research Center) using a TissueLyser (Qiagen) at 25 Hz for 7 min. After centrifugation at 12000*g* for 10 min at 4°C, 10% bromochloropropane was added and samples were centrifuged at 12000*g* for 15 min at 4°C for phase separation. The upper, aqueous phase was collected, and RNA was precipitated using cooled 70% ethanol. RNeasy columns (Qiagen) were used for RNA purification according to the manufacturer's instructions. RNA purity and concentration was measured with the NanoDrop system (Witec GmbH). Samples were stored at −80°C until use.

cDNA was synthesized from 600 ng RNA using TaqMan® Reverse Transcription reagents (Applied Biosystems, Invitrogen) with random hexamer primers. Real-time PCR was performed using 6 ng cDNA and TaqMan Master Mix (Applied Biosystems) using a Quant Studio 6 Flex machine (Applied Biosystems). Genes of interest were detected using TaqMan Gene Expression Assays (Applied Biosystems) for angiopoietin 1 (Hs00181613_m1), CD146/MCAM (Hs00174838), NG-2/CSPG 4 (Hs00426981_m1), connexin 43 (Hs00748445_s1), collagen IV (Hs00266237_m1), platelet-derived growth factor receptor *β*1 (PDGFR*β*1) (Hs00182163_m1), and Tie 2/Tek receptor (Hs00176096). Vascular endothelial growth factor A (VEGF A) was detected using forward primer 5′-GCC CAC TGA GGA GTC CAA CA-3′, reverse primer 5′-TCCTATGTG CTG GCC TTG GT-3′, and probe 5′-CAC CAT GCA GAT TAT GCG GAT CAA ACC T-3′ (Microsynth). As an endogenous control, human 18s (Hs99999901_m1, ThermoFisher Scientific) was used. All samples were measured in duplicates, and data are presented relative to day 0 using the comparative ∆∆CT method.

### 2.7. Histology

Histological analysis was performed of gels with mono- and cocultures after 14 days of incubation. Gels were washed once with PBS and then placed in cryomolds with cryocompound Tissue Freezing Medium (Jung). After 15 min of incubation, samples were snap frozen in isopentane and stored at −20°C until use.

Snap-frozen samples were cut in 20 *μ*m thick slices. For immunohistology, cuts were fixed in 70% methanol for 15 min, rehydrated in dH_2_O, and incubated for 30 min in 99.5% methanol with 30% H_2_O_2_ to block endogenous peroxidase activity. After blocking for unspecific antibody binding with horse serum (dilution 1 : 20) at room temperature for 60 min, the primary antibodies connexin 43 (ThermoFisher Scientific) and CD146 (abcam) were put onto the slides (final concentration: 5 *μ*g/*μ*l connexin 43; 1 *μ*g/*μ*l CD146) and incubated for 30 min. Controls were incubated with PBS containing 0.1% Tween (PBST). Afterwards, slides were washed three times with PBST and incubated for 60 min with a secondary anti-mouse antibody (Vectastain ABC kit) at a dilution of 1 : 200. Another washing step (3 × PBST) was performed, the ABC complex of the Vectastain ABC Elite kit was applied, and slides were incubated for 30 min. After washing 3 × with PBST, slides were incubated with ImmunPACT DAB in the dark for 4 min, and after several water changes in dH_2_O, slides were covered with Prolong Gold antifade reagent with DAPI (ThermoFisher Scientific). Slides were stored at room temperature until observation, and pictures were taken using an Axioplan2 microscope equipped with an AxioCamHRc camera and AxioVision software (Zeiss).

### 2.8. Statistics

All values are shown as mean ± standard error of the mean (SEM). Statistical analysis of data was performed with GraphPad Prism 7 software. Normal distribution of data was proven using the Shapiro-Wilk normality test. Statistical differences between experimental groups were tested by 2-way ANOVA and Tukey's post hoc test for multiple comparisons.

## 3. Results

### 3.1. Shrinkage of PRP Gels with Encapsulated MSCs

**Figure 2:**
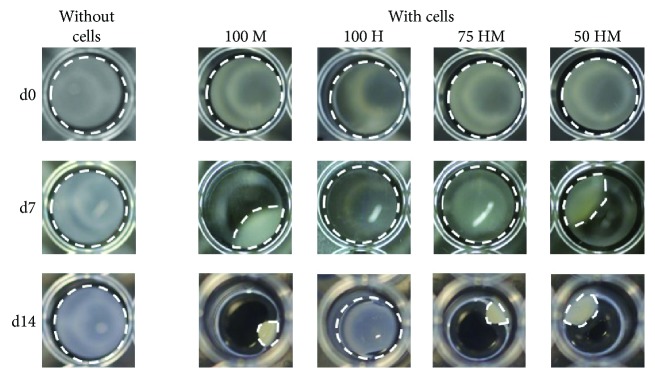
MSC mediated shrinkage of PRP gels. PRP gels (120 *μ*l) were seeded either with or without cells in a 96-well plate. Different types of cells were encapsulated (seeding density: 2.5 × 10^3^ cells/*μ*l gel): 100% MSCs (100 M), 100% HUVECs (100 H), 75% HUVECs–25% MSCs (75 HM), 50% HUVECs–50% MSCs (50 HM). Shown are representative pictures from day 0 (d0), day 7 (d7), and day 14 (d14). White dashed lines indicate the outline of gels. Shrinkage was only observed when MSCs were present in gels.

Figure 2 shows representative pictures of PRP gels with and without cells taken at different time points during the culture period. PRP gels without cells retained their original shape, while shrinkage occurred in cell-containing gels. After one week, most pronounced shrinkage could be observed in gels with MSCs monocultures, where gels shrinked to nearly half of their original volume. Moreover, gels containing MSC-HUVEC cocultures (50 HM) demonstrated a reduced size, whereas HUVEC-containing gels were not affected. After two weeks, the shrinkage was still ongoing, however, at a slower rate. Again, shrinkage was most pronounced in gels containing the highest concentrations of MSCs, suggesting that this effect was dependent on the number of encapsulated MSCs.

### 3.2. Stable Cellular Networks Are Present in HUVEC-MSC Cocultures after 14 Days

**Figure 3:**
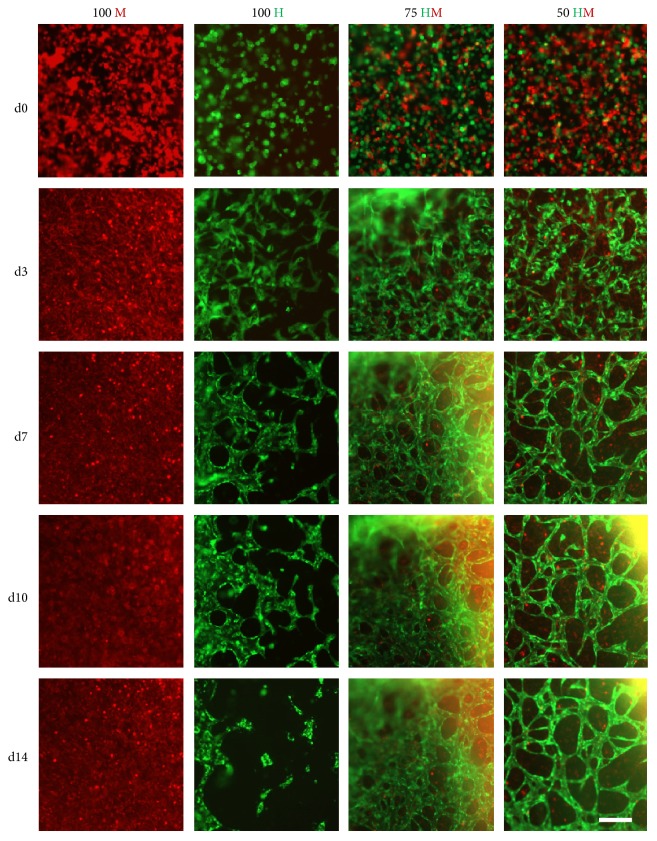
Time-lapse pictures of HUVEC and MSC mono- and cocultures. GFP-positive HUVECs (green) and PKH26 Red prestained MSCs were encapsulated in PRP (seeding density: 2.5 × 10^3^ cells/*μ*l gel) and time-lapse microscopy ran for two weeks. Four different cell proportions were seeded: 100% MSCs (100 M, first column), 100% HUVECs (100 H, second column), 75% HUVECs–25% MSCs (75 HM, third column), and 50% HUVECs–50% MSCs (50 HM, fourth column). Representative pictures of five time points are shown: day 0 (d0), day 3 (d3), day 7 (d7), day 10 (d10), and day 14 (d14). Cellular organization towards formation of tube-like networks starting from day 3 was observed in mono- and cocultures in PRP over time but not in the condition of 100% MSCs. After one week, a complex cellular network could be detected in both cocultures which was still apparent after two weeks. Cellular networks in HUVEC monocultures disintegrated after 10 days. Scale bar = 200 *μ*m.

**Figure 4:**
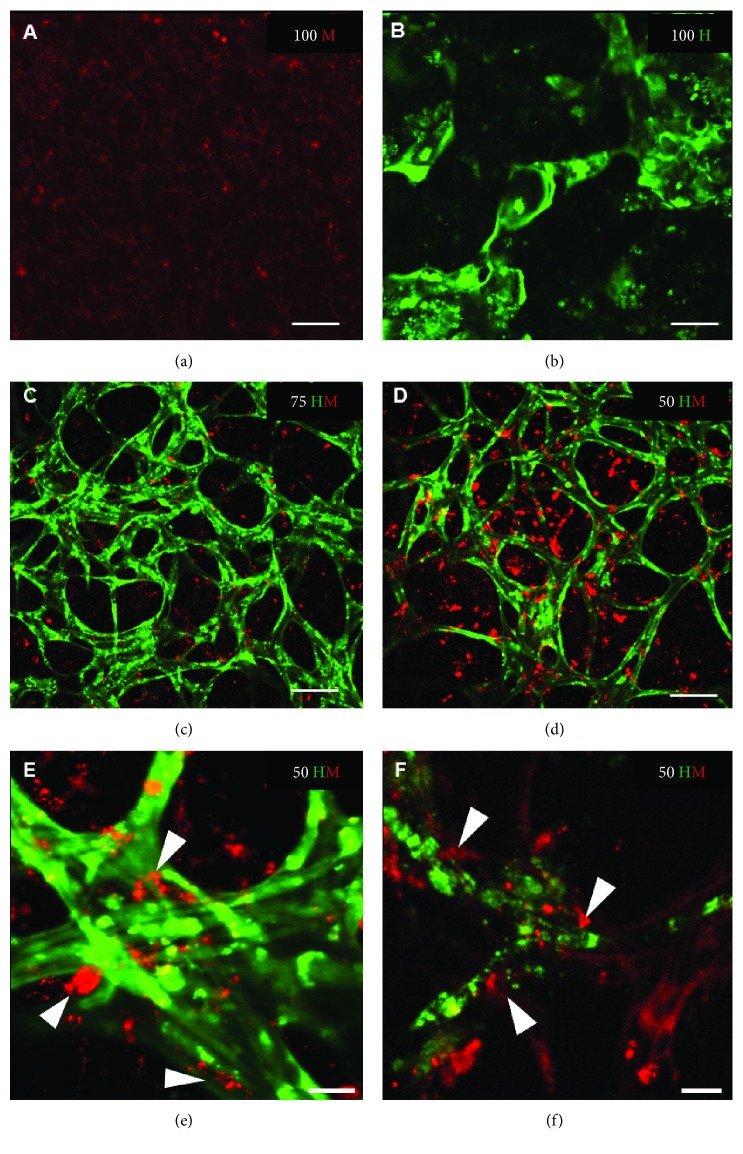
High-resolution images of HUVEC and MSC mono- and cocultures after 2 weeks. Representative images are shown from mono- and cocultures at day 14 after seeding (seeding density: 2.5 × 10^3^ cells/*μ*l gel) with (a) 100% MSCs (100 M); (b) 100% HUVECs (100 H); (c) 75% HUVECs–25% MSCs (75 HM); and (d–f) 50% HUVECs–50% MSCs (50 HM). Network formation occurred in 100 H and both cocultures but was only stable in the presence of MSCs which integrate in cellular networks as demonstrated in e and f (white arrow heads). Scale bar = 100 *μ*m (a–d), 25 *μ*m (e, f).

Cellular organization within PRP gels was observed by time-lapse microscopy (Figure 3). In HUVEC monocultures, the formation of networks was present as early as 3 days after seeding. Branching and complexity of the networks increased until day 10. Afterwards, structures became disorganized. In contrast to HUVEC monocultures, no networks could be detected in MSC monocultures. In both cocultures (75 HM and 50 HM), cellular networks were observed (Figure 3). Cells were well organized in networks already after 3 days. Defined networks could be observed after one week of culture. In contrast to the monocultures, these networks seemed to be stable for 2 weeks. To note, the 3D nature of cellular networks often hindered a high-quality imaging. To address this issue, high-resolution images were taken with a confocal microscope at the end of the study after 14 days (Figure 4). The findings were in line with the observations from time-lapse imaging (Figure 3). However, high magnification images revealed the contribution of MSCs to the networks (Figures 4(e) and 4(f)).

**Figure 5:**
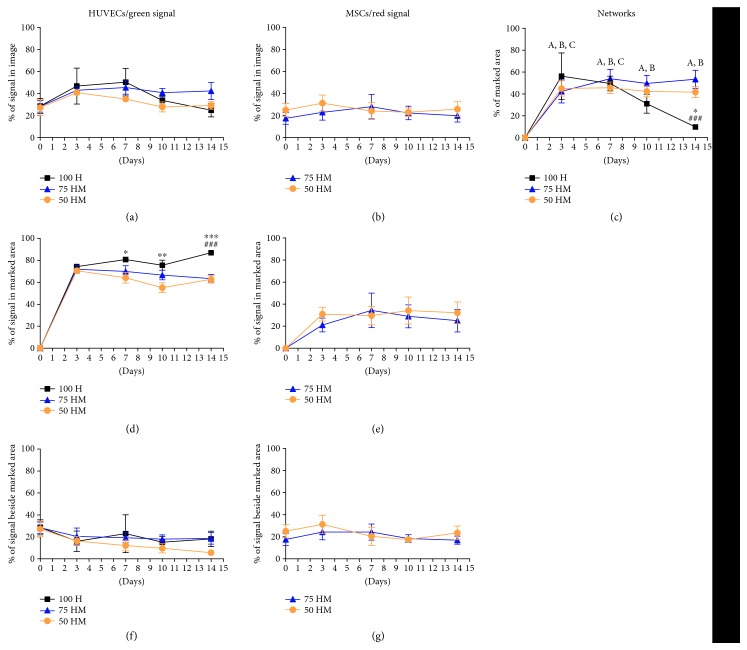
Analysis of cellular networks. Image analysis was performed of mono- and cocultures (100% HUVECs (100 H), 75% HUVECs–25% MSCs (75 HM), and 50% HUVECs–50% MSCs (50 HM)) in PRP (seeding density: 2.5 × 10^3^ cells/*μ*l gel) (*n* = 3). Networks were measured by manually marking cellular networks. The relative percentage of prestained green (HUVECs) and red (MSCs) cells was investigated for day 0, day 3, day 7, day 10, and day 14 in the following regions: relative percentage of green or red signal in the entire image (a, b), relative percentage of marked network area (c), relative percentage of green or red signal in marked network area (d, e), and the relative percentage of green or red signal beside marked network area (f, g). For 100 H monocultures, networks decreased after one week whereas in both cocultures, networks were present and stable after two weeks (c). Cellular networks were mainly made out of HUVECs (d), with a minor contribution of MSCs (e). Two-way ANOVA with Tukey's post hoc test for multiple comparison was applied to test for significant differences over time (compared to day 0: (A) 50 HM *p* < 0.01 (all time points); (B) 75 HM *p* < 0.01 (day 3) and *p* < 0.001 (day 7–14); and (C) 100 H *p* < 0.001 (day 3) and *p* < 0.01 (day 7)) and between different culture conditions (^∗^*p* < 0.05, ^∗∗^*p* < 0.01, ^∗∗∗^*p* < 0.001 compared to 50 HM, ^###^*p* < 0.001 compared to 75 HM). *n* = 3.

Based on time-lapse pictures, image analysis was performed to investigate further the formation of cellular networks in different culture conditions (Figure 5). First, we measured the relative percentage of green and red fluorescence in the entire image, representing encapsulated HUVECs and MSCs, respectively (Figures 5(a) and 5(b)). A slight trend of increase in measured green fluorescent signal in the entire image was detected in all conditions by day 3 (Figure 5(a)), raising from 28.7 ± 6.9% to 46.9 ± 16.4%, from 28.4 ± 5.2% to 43 ± 3.3%, and from 27.3 ± 7% to 40.9% in 100 H, 75 HM, and 50 HM cultures, respectively (all *p* > 0.05). From there on, signals remained at a constant level, or even decreased to baseline levels with 25 ± 6%, 42 ± 7.9%, and 29 ± 3.5% at day 14 for 100 H, 75 HM, and 50 HM cultures, respectively. A similar pattern was observed for the red fluorescence signal, that is, the MSCs (Figure 5(b)). These findings demonstrate that over the course of the experiment, cells were apparent at least in a similar concentration as at day 0, suggesting that PRP offers an appropriate environment for cells over a time of 2 weeks.

Next, we analyzed the efficiency of network formation in the different culture conditions (Figure 5()(c)). Results showed that the main parts of networks were built within the first three days in all conditions, with networks covering 56.3 ± 21.3%, 42.4 ± 10.6%, and 45 ± 7% of the entire image area in 100 H, 75 HM, and 50 HM cultures, respectively (all *p* < 0.01 compared to day 0). For 75 HM and 50 HM cocultures, a stable high area of cellular networks which remained constant over time (*p* < 0.01 compared to day 0 for all time points) was observed with only minimal differences between both culture conditions. While the speed of initial network formation as well as the percentage of formed networks in HUVEC cultures were comparable to cocultures until day 7 (*p* < 0.01 compared to day 0 for day 3 and day 7), networks significantly decreased after 10 days, revealing similar concentrations as on day 0 (*p* > 0.05). After 14 days, networks were only observed in the cocultures with MSCs (53.5 ± 8.3% for 75 HM and 41.7 ± 5% for 50 HM, versus 9.8 ± 1% for 100 H), which led to the suggestion that MSCs play a fundamental role in the stabilization processes of newly formed structures.

Finally, we investigated the contribution of HUVECs (Figure 5(d)) or MSCs (Figure 5(e)) to the marked network area. Analysis revealed that most HUVECs were involved in the formation of networks in all conditions with a relative contribution to the networks ranging from 55% to 87% depending on the culture condition and time point. The fast increase of the green signal measured in networks (Figure 5(d)) affirms again the speed of network formation in all conditions. Although cellular networks were less stable in 100 H monocultures, the contribution of HUVECs to the networks in these cultures was higher compared to cocultures reaching statistical significance from day 7 on (day 7: 100 H 80.9 ± 0.2% versus 50 HM 64.2 ± 4.8% (*p* < 0.05); day 10: 100 H 75.7 ± 4.6% versus 50 HM 55.2 ± 4.6% (*p* < 0.01); and day 14: 100 H 87 ± 0.9% versus 75 HM 63.4 ± 3.8% (*p* < 0.001) versus 50 HM 62.8 ± 3.3% (*p* < 0.001)). With 21–34% of the network area covered by red fluorescence signal, MSCs contributed to the networks as well, however, to a lesser extent as HUVECs.

The fluorescence signal detected outside the marked network area (Figures 5(f) and 5(g)) reflects the timely recruitment of cells to the networks at a merely constant total cell number.

### 3.3. Analysis of Angiogenic Gene Expression in PRP Gels Containing Cellular Networks

**Figure 6:**
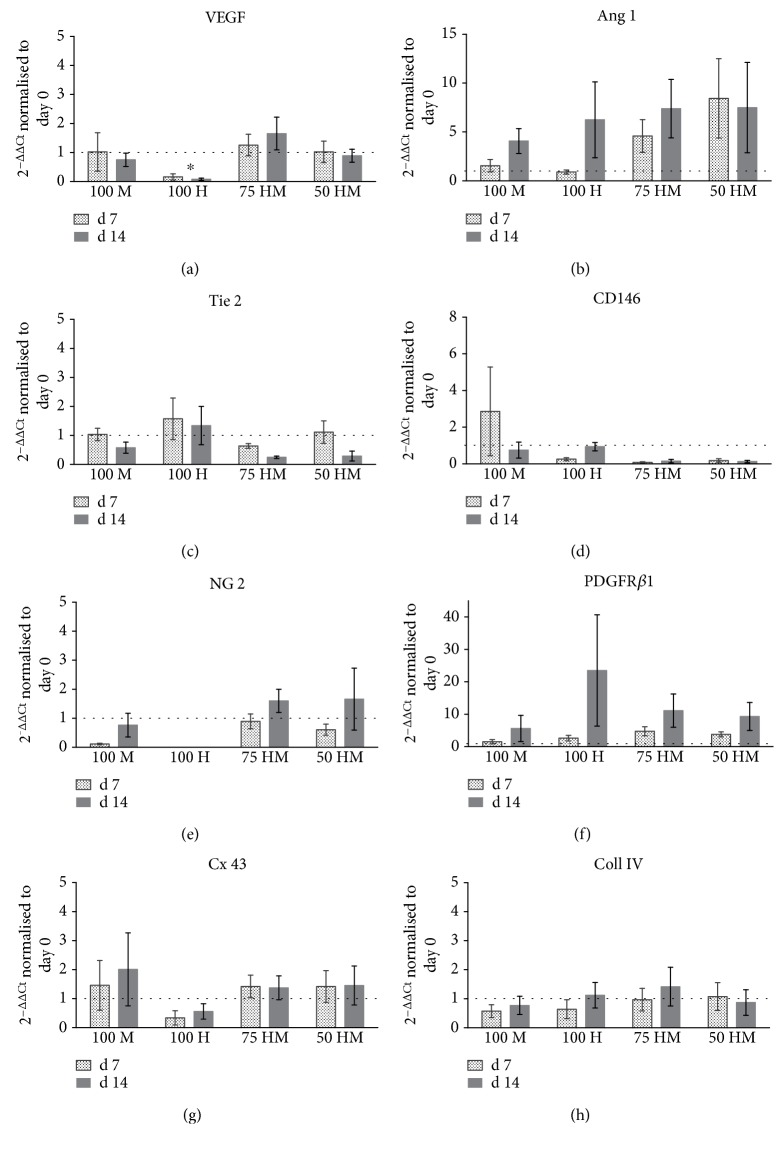
Gene expression of HUVEC and MSC mono- and cocultures in PRP at days 7 and 14. Gene expression (normalized to 18s expression) at day 7 and day 14 is displayed as fold change to day 0. Dashed lines indicate a fold change of 1, that is, unchanged gene expression. MSC monocultures (100 M), HUVEC monocultures (100 H), and cocultures 75% HUVECs–25% MSCs (75 HM) and 50% HUVECs–50% MSCs (50 HM) were investigated (seeding density: 2.5 × 10^3^ cells/*μ*l gel). (a) Gene expression of VEGF, showing downregulation in 100H cultures. (b) An upregulation of angiopoetin-1 (Ang 1), a crucial growth factor of angiogenic processes, was detected in all conditions at day 14 as well as in the cocultures on day 7. (c) Gene expression of Tie 2, one of the receptors binding Ang 1. (d–f) Depicted is the expression of pericyte markers CD146, NG 2, and PDGFR*β*1, respectively. CD146 was upregulated at day 7 in MSC monocultures (d). At day 7, both cocultures showed a higher NG 2 expression compared to monocultures (e). PDGFR*β*1 upregulations were detectable in all cultures at day 14 (f). MSCs upregulated connexin 43 (g), indicating the formation of gap junctions, whereas no differential regulation was observed for collagen IV (h). Two-way ANOVA with Tukey's post hoc test for multiple comparison was applied to test for differences between experimental groups; ^∗^*p* < 0.05. *n* = 3.

All PRP gels were analyzed for the gene expression related to angiogenesis and vascularization at day 7 and day 14 (Figure 6). First, we measured the expression of VEGF (Figure 6(a)). Although VEGF plays an important role in terms of new vessel formation, data shows no upregulation of VEGF gene expression in PRP gels. However, in contrast to HUVEC monocultures, where VEGF expression was downregulated, gene expression remained stable in cocultures (100 H versus 75 HM *p* < 0.05 at day 14) and MSC monocultures. Of note, the lack of upregulation of VEGF might be related to the fact that both the feeding medium EGM-2 and PRP itself contain high levels of VEGF.

Another main growth factor involved in angiogenic processes is angiopoietin 1, which showed a trend of upregulation in all conditions (ranging from a fold change of 4 to 7 in the different culture conditions, *p* > 0.05) at day 14 (Figure 6(b)). In line with the efficient formation of cellular networks in cocultures, angiopoietin 1 was specifically upregulated in these cultures at day 7 (75 HM fold change: 4.6 ± 1.7; 50 HM fold change 8.4 ± 4) which was however not statistically significant (*p* > 0.05). In contrast to these findings, the angiopoietin receptor Tie 2 did not show a similar pattern as angiopoietin 1 (Figure 6(c)). While Tie 2 gene expression was unchanged in most culture conditions, a slight downregulation was detected in cocultures at day 14 (*p* > 0.05).

Pericytes, which are closely related to MSCs, are involved in the stabilization of newly formed vessels. Therefore, we analyzed the expression of CD146, NG-2, and PDGFR*β*1 as putative pericyte markers (Figures 6(d)–6(f)). For CD146, gene expression of MSC monocultures revealed a slight upregulation on day 7 (*p* > 0.05, Figure 6(d)). In contrast, NG-2 gene expression, which was completely absent in HUVEC monocultures, was downregulated in the MSC monoculture and tended to be stable in cocultures (fold change: 100 M 0.1 versus 75 HM 0.9 ± 0.3 and 50 HM 0.6 ± 0.2, all *p* > 0.05). Besides the lack of NG-2 expression in HUVEC monocultures, no differences in NG-2 gene regulation were observed at day 14. A trend of upregulation was apparent for PDGFR*β*1 at day 14 with large variations between different cell donors (Figure 6(f)). The highest upregulation of PDGFR*β*1 was detected in HUVEC monocultures (fold change 23.5 ± 17.2, *p* > 0.05).

Apart from growth factors and their receptors, cell-to-cell contacts, as well as basal membrane proteins play an important role in angiogenesis. Connexin 43 is involved in gap junction cell-to-cell-contacts. A tendency for a slight upregulation of connexin 43 was observed in MSC monocultures (Figure 6(g)), whereas no changes were apparent in collagen IV gene expression, a basal membrane protein (Figure 6(h)).

### 3.4. Immunohistological Analysis of CD146 and Connexin 43

**Figure 7:**
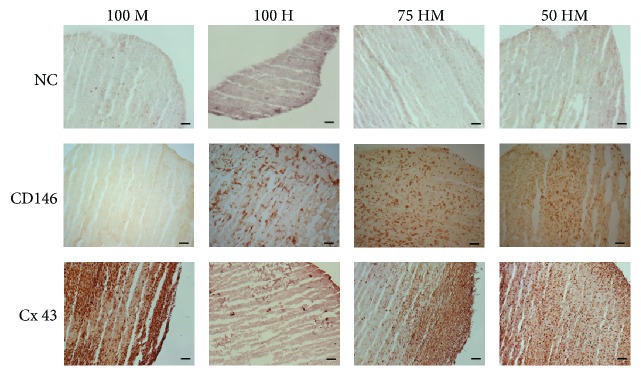
Immunohistology for CD146 and connexin 43 (Cx 43) of HUVEC and MSC mono- and cocultures in PRP at day 14. Immunohistology was performed on cryosections of mono- and cocultures with 100% MSCs (100 M), 100% HUVECs (100 H), 75% HUVECs–25% MSCs (75 HM), and 50% HUVECs–50% MSCs (50 HM) (seeding density: 2.5 × 10^3^ cells/*μ*l gel). Negative controls (NC) are shown in the first row. In comparison to the NC, positive signals of CD146 were detectable in the presence of HUVECs, whereas connexin 43 protein levels were only apparent in a mono- or coculture with MSCs. Scale bars = 100 *μ*m.

To evaluate specific markers on protein level, immunohistology was performed on cryosections for CD146, a pericyte marker, and connexin 43, a cell-to-cell contact protein (Figure 7()). CD146 was detectable in the presence of HUVECs whereas MSCs alone showed similar results than negative controls indicating that MSC alone did not express CD146 at a detectable level in 3D culture. Connexin 43 was only present in mono- or cocultures containing MSCs.

## 4. Discussion

In the context of musculoskeletal regeneration, for example, bone defects, limitations in the healing process often occur due to an insufficient blood supply at the defect site. Revascularization involves angiogenic sprouting of vessels from the surrounding tissue, which frequently does not occur in an appropriate time, as the ingrowth of vessels is a long-term process [3]. Therefore, prevascularized implants have been suggested as a promising treatment strategy [6–15]. The aim of this study was to create an autologous cell delivery system for prevascularized constructs within PRP hydrogels.

In the present study, we showed that PRP is indeed a suitable delivery system for HUVECs, MSCs, and cocultures of both. Cells survived for a period over 14 days which is in line with our previous work [33] demonstrating the viability of MSCs encapsulated in PRP gels for one week.

We noticed that, particularly in the presence of MSCs, PRP gels shrinked over time. Upon *in vivo* implantation, shrinkage of gels and the herewith associated volume loss may be a limitation for the use of PRP as a cell delivery system. However, shrinkage of gels was not directly related to the formation of networks. In fact, cell assembly to tube-like structures occurred as early as 3 days in cell culture, and thereby, this preceded the significant reduction of gel volume observed after 7 days. This indicates that early implantation of prevascularized PRP gels would be desirable. Short *in vitro* culture periods would also be most convenient for clinical applications. In addition, mesenchyme-driven condensation of soft matrix has been reported as an important step in the formation of organ buds and the regeneration of functional vascularized tissues [47]. Therefore, the observed shrinkage, which in line with findings from Takebe et al. was mainly driven by MSCs, may be considered as natural step in matrix remodeling.

We observed that PRP gels with cocultures of HUVECs and MSCs formed networks as early as 3 days after cell encapsulation, which were still stable after 2 weeks. This fast network formation might be stimulated by the growth factors secreted from PRP gels [33]. Indeed, since cells were resuspended in PRP upon activation with thrombin, they were immediately exposed to the growth factor release.

In agreement with earlier studies [10, 11, 48], cells (HUVECs and MSCs) took advantage of coculturing. In contrast, no stable networks could be detected in HUVEC monocultures. The maturation of new blood vessels requires the recruitment of mural cells (pericytes or smooth muscle cells) [49] to be stable in the long term. Pericytes are found as single cells, distributed at discontinuous intervals along the basal membrane, which they share with endothelial cells [50]. During angiogenesis, pericytes are stimulated for migration and proliferation before they are recruited to new vessels to contribute to stability. Recently, we have investigated the plasticity of MSCs and their ability to differentiate into a pericyte-like phenotype [51]. This study showed that MSCs upregulated the expression of pericyte markers such as NG-2 and CD146 when they are in direct coculture with bone marrow-derived EPCs [51]. Similarly, Goerke and colleagues showed differentiation of MSCs towards a smooth muscle cell phenotype when coincubated with blood derived EPCs [52]. In line with these findings, MSC supplementation has been proven to promote the formation of stable vessels in various *in vitro* and *in vivo* studies [9, 10, 13, 15, 23, 53]. For example, our previous work showed that tissue-engineered constructs composed of EPC-MSC cocultures delivered in PU scaffold in the presence of PRP, promoted vascularization upon subcutaneous implantation [15]. Furthermore, we observed that MSCs indeed display a pericyte-like phenotype in these cocultures [23].

Pericytes, best defined based on their *in vivo* localization, express several surface markers including CD146 [54, 55], NG-2 [56], *α*-SMA [57], and PDGFR*β*1 [58], which is however dependent on their localization [59]. In the present study, we analyzed the gene expression of CD146, NG-2, and PDGFR*β*1. Results gave evidence for a differentiation of MSCs into a pericyte-like phenotype as described above. This was particularly obvious by a much higher expression of NG-2 in cocultures in comparison to HUVEC monocultures. Moreover, we detected a general upregulation of PDGFR*β*1 in all cultures. The PDGF*β*/PDGFR*β* pathway is critically important for the expansion of the pericyte population and pericyte migration along growing vessels [60]. Therefore, upregulation of its gene expression in cocultures might reflect the differentiation and recruitment of MSCs towards the vessel-like structures in PRP gels. Strikingly, also HUVEC monocultures showed an upregulation of PDGFR*β* gene expression. Although, endothelial cells usually do not express PDGFR*β*1 in high quantities and the relevance of this receptor is unclear in resting endothelium, the current literature provides evidence that PDGFR*β* is upregulated under circumstances of angiogenesis [61–63]. Therefore, we hypothesize that PDGFR*β* upregulation in HUVEC cultures might be caused by the formation of tube-like networks and possibly might be a response to the absence of supporting mural cells. Taken together, in the current study, we only detected a relatively mild increase in the expression of pericyte marker missing statistical significance. Since changes in gene expression are generally transient, it is likely that we might have missed certain changes because only two time points were assessed.

Various signaling pathways are involved in angiogenesis, and it would be of most interest to perform a broad screening of factors involved in the proangiogenic functions of PRP. In this first evaluation of PRP gel as a proangiogenic cell delivery system, we however focused on few key players in angiogenesis; future studies will thoroughly investigate the mechanism of tube-like structure formation in PRP gels. One major growth factor is angiopoietin 1 which acts as a survival signal for endothelial cells and further promotes vascular stabilization by facilitating pericyte recruitment [64, 65]. Here, we show a tendency of angiopoietin 1 upregulation, with the highest upregulations in cocultures of HUVECs and MSCs on day 7 and day 14. However, Tie 2, which is constitutively activated by basal angiopoietin 1 expression [66], was not expressed. An explanation might be the general high expression of Tie 2 on surfaces of mature endothelial cells (HUVECs) which could be seen in gene expression in monoculture conditions. In addition, Tie 2 expression may be delayed to angiopoietin 1 and therefore not be seen at the analysis time points.

In respect of angiogenesis, VEGF is another crucial growth factor for inducing new vessel formation. The fact that the applied growth medium EGM-2 as well as PRP itself already contains VEGF might be a reason why we could not find an upregulation of VEGF gene expression. Moreover, previous studies reported comparable findings since it was shown that new vessel formation is not generally associated with changes in VEGF expression [67, 68].

Moreover, we investigated the expression of the specific cell-to-cell contact protein connexin 43 and found that it was expressed in dependence of MSCs, especially on day 14. Connexin 43 is part of gap junctions and expressed on endothelial cells [69]. In addition, it was previously shown that MSCs overexpressing connexin 43 promote neovascularization [70]. Furthermore, Wang et al. proposed that connexin 43 may act as a potential target for improving the therapeutic efficacy of MSC transplantation. In line with that, we found MSC-dependent expression of connexin 43 on both gene and protein levels. Since vascular network structures were only stable in dependence of MSCs, this leads to the hypothesis that connexin 43 expressed on MSCs may contribute to stabilization processes of cellular networks.

In this study, we present for the first time an autologous delivery system for prevascularized constructs. PRP can easily be harvested by drawing blood which may facilitate the next steps to clinical applications. The fact that PRP is more and more used in clinical studies [29, 32, 71] emphasizes its importance in musculoskeletal healing processes. We demonstrate that PRP has a great potential to support neovascularization, which is a major clinical challenge, particularly in the context of bone repair. While the study confirmed PRP as autologous and proangiogenic cell delivery system, the endothelial cell source (HUVECs) used here cannot be obtained autologously. However, at this stage, we focused on the investigation of these proangiongenic properties of PRP; future studies will address the use of autologous cell sources such as peripheral blood or bone marrow-derived EPCs.

Since the current study is an *in vitro* experiment, it cannot foresee how cellular networks will sustain in an *in vivo* environment and how cell-to-cell communication and a supposedly fast *in vivo* degradation of PRP will impact the stability of tubular structures as well as the anastomosis to the host vascular system. In addition, future studies should be conducted to optimize the system for the application *in vivo*. These studies should address the implantation technique and time point as well as cell seeding densities. Eventually, it will be interesting to test whether a delivery of cells is necessary or if the chemoattractive and proangiogenic properties of PRP themselves may be sufficient to support neovascularization.

## 5. Conclusion

To our knowledge, this is the first study to report on an autologous cell delivery system for the delivery of prevascularized structures. Our study shows stable formation of cellular networks by combining HUVECs and MSCs in one culture system. The contribution of MSCs, possibly differentiating into a pericyte-like phenotype, may be crucial for the stabilization of cellular networks.

In conclusion, we demonstrate that PRP can be used as an autologous, proangiogenic cell delivery system promoting early neovascularization.
